# The Clinical Heterogeneity of Spinal Muscular Atrophy with Respiratory Distress Type 1 (SMARD1)—A Report of Three Cases, Including Twins

**DOI:** 10.3390/genes15080997

**Published:** 2024-07-30

**Authors:** Alicja Leśniak, Marta Glińska, Michał Patalan, Iwona Ostrowska, Monika Świrska-Sobolewska, Kaja Giżewska-Kacprzak, Agata Kotkowiak, Anna Leśniak, Mieczysław Walczak, Robert Śmigiel, Maria Giżewska

**Affiliations:** 1 Department of Pediatrics, Endocrinology, Diabetology, Metabolic Diseases and Cardiology of the Developmental Age, Pomeranian Medical University in Szczecin, 71-252 Szczecin, Poland; alicja.lesniak@pum.edu.pl (A.L.); mglinskamd@gmail.com (M.G.); maria.gizewska@pum.edu.pl (M.G.); 2 Department of Pediatric and Oncological Surgery, Urology and Hand Surgery, Pomeranian Medical University in Szczecin, Prof. Tadeusz Sokołowski University Clinical Hospital No. 1, 71-252 Szczecin, Poland; 3 Department of Pediatrics, Endocrinology, Diabetology and Metabolic Diseases, Wroclaw Medical University, 50-368 Wroclaw, Poland

**Keywords:** SMARD1, DSMA1, HMNR VI, *IGHMBP2*, diaphragm paralysis, progressive distal muscle weakness

## Abstract

Spinal muscular atrophy with respiratory distress type 1 (SMARD1; OMIM #604320, ORPHA:98920) is a rare autosomal recessive congenital motor neuron disease. It is caused by variants in the *IGHMBP2* gene. Clinically, it presents with respiratory failure due to diaphragmatic paralysis, progressive muscle weakness starting in the distal parts of the limbs, dysphagia, and damage to sensory and autonomic nerves. Unlike spinal muscular atrophy (SMA), SMARD1 has a distinct genetic etiology and is not detected in the population newborn screening programs. Most children with SMARD1 do not survive beyond the first year of life due to progressive respiratory failure. Artificial ventilation can prolong survival, but no specific treatment is available. Therapy focuses on mechanical ventilation and improving the patient’s quality of life. Research into gene therapy is ongoing. We report three female patients with SMARD1, including twins from a triplet pregnancy. In twin sisters (patient no. 1 and patient no. 2), two heterozygous variants in the *IGHMBP2* gene were identified: c.595G>C/p.Ala199Pro and c.1615_1623del/p.Ser539_Tyr541del. In patient no. 3, a variant c.1478C>T/p.Thr493Ile and a variant c.439C>T/p.Arg147* in the *IGHMBP2* gene were detected. Our findings underscore the variability of clinical presentations, even among patients sharing the same pathogenic variants in the *IGHMBP2* gene, and emphasize the importance of early genetic diagnosis in patients presenting with respiratory failure, with or without associated diaphragmatic muscle paralysis.

## 1. Introduction

Spinal muscular atrophy with respiratory distress type 1 (SMARD1; OMIM #604320, ORPHA:98920), also known as autosomal recessive distal hereditary motor neuronopathy-1 (HMNR1), is a rare congenital motor neuron disease [1,2]. Mellins et al., in 1974, and subsequently Bertini et al., in 1989, described groups of patients exhibiting respiratory failure secondary to diaphragmatic paralysis with decreased muscle strength in distal parts of the limbs [3,4]. They classified this condition as an infantile form of spinal muscular atrophy (SMA; OMIM #253300) [5]. However, in 1999, Grohmann et al. demonstrated a different genetic background for the disorder, leading to the recognition of a new distinct clinical entity [6].

SMARD1 arises from homozygous or compound heterozygous variants in the *IGHMBP2* gene (600502) located on chromosome 11q13.2-q13.4 [7]. Reported variants include missense, nonsense, splicing, small intragenic deletions, insertions causing frameshift, and more significant aberrations encompassing the region mentioned above. Biallelic variants in *IGHMBP2* can also manifest as axonal Charcot–Marie–Tooth disease type 2S (CMT2S; OMIM #616155) [8,9].

The *IGHMBP2* gene consists of 15 exons and encodes the immunoglobulin helicase μ-binding protein 2 (IGHMBP2). This protein functions as an ATP-dependent helicase with 5’ to 3’ polarity, unwinding RNA and DNA duplexes in an ATP-dependent reaction, the process essential in various stages of cell division [10]. Pathogenic variants in this gene have also been implicated in a broad spectrum of axonal neuropathies, including damage to alpha motor neurons in the anterior horns of the spinal cord [9,11,12,13].

The exact prevalence of the disease is not known. However, according to Orphanet, it is estimated to be <1/1,000,000 [14]. By now, approximately 150 patients with this diagnosis have been described [13,15,16].

Three forms of SMARD1 are distinguished based on the age of initial symptom onset: (1) the early-onset form, associated with the most severe course and prognosis, where symptoms appear before 3 months of age; (2) the typical/classic form, where initial symptoms are observed between 3 and 12 months of age; and (3) the late-onset form, characterized by a relatively mild course, with symptoms appearing after the first year of life [17].

The typical clinical presentation of SMARD1 includes progressive respiratory failure with diaphragmatic paralysis, progressive muscle weakness starting in the distal parts of the limbs, difficulties swallowing, and damage to sensory and autonomic nerves. Additionally, from birth, features such as weak crying, poor sucking reflex, decreased muscle tone, and, in subsequent weeks, breathing difficulties (wheezing, recurrent episodes of breathlessness or apnea, cyanosis) and absent deep tendon reflexes are noted. As muscle strength diminishes in the feet and later in the fingers, joint contractures develop. An additional characteristic feature is the atrophy of small hand muscles and the accumulation of adipose tissue at the proximal phalanges of the fingers (fatty pads). Pronounced kyphosis/scoliosis or foot deformities are often present [17,18]. Unlike in SMA, chest deformities resulting from intercostal muscle atrophy are not observed [19]. Although limited data are used to evaluate the cognitive functions of patients with SMARD1, it appears that the majority fall within the normal range [13].

The average lifespan of patients without mechanical ventilation is approximately nine months [13]. It is difficult to estimate the prognosis of patients on respiratory support, though it has significantly improved due to the better quality of home ventilation. Currently, the main causes of death in patients with SMARD1 are the progressive dysfunction of the autonomic system and complications of respiratory therapy (e.g., sepsis, pneumonia) [1].

At present, no causal treatment is available, so therapy is focused on appropriate mechanical ventilation, physiotherapy, and enhancing patients’ quality of life. Investigation into gene therapy remains ongoing [17,20]. We present three cases of patients with SMARD1, including twin sisters. In twin sisters (patient no. 1 and patient no. 2), two heterozygous variants in the *IGHMBP2* gene were identified: c.595G>C/p.Ala199Pro and c.1615_1623del/p.Ser539_Tyr541del. In patient no. 3, a variant c.1478C>T/p.Thr493Ile and a variant c.439C>T/p.Arg147* in the *IGHMBP2* gene were detected.

## 2. Case Description

### 2.1. Patient No. 1 (Twin I)

The female infant was born from the first triplet pregnancy to unrelated parents. The family history included hypothyroidism, hypercholesterolemia, and gout in the father as well as a hiatal hernia in the mother. The healthy brother (triplet I) exhibited normal development.

The girl was born as triplet II, delivered prematurely at the 33rd week of gestation via cesarean section in a fair general condition with Apgar scores of 7/8/8, with a birth weight of 1000 g (−2.57 SD), a body length of 37 cm (−2.4 SD), and an occipital–frontal circumference (OFC) of 25 cm (−3.33 SD). She presented features of morphological immaturity and fetal growth restriction (FGR). The neonatal period was complicated by respiratory distress, necessitating non-invasive respiratory support (infant flow and continuous positive airway pressure (CPAP)) and surfactant administration. The patient also suffered from severe anemia requiring multiple blood transfusions, transient bradycardia, apnea, feeding difficulties, muscle hypotonia, and hypothyroidism.

At the age of 3 months, she was hospitalized due to apnea episodes observed at home. By then, an infectious etiology was ruled out. At four months of age, severe respiratory syncytial virus (RSV)-associated pneumonia required admission to a pediatric pulmonology department and, subsequently, a pediatric intensive care unit (PICU). A right-sided pneumothorax was revealed on the chest X-ray without evidence of diaphragmatic paralysis. Treatment involved mechanical ventilation, pleural drainage, antibiotic therapy, and nutritional support via intragastric tube. Neurological assessment revealed muscle hypotonia, predominantly in the lower limbs. Brain MRI showed delayed white matter myelination and mild ventricular enlargement. However, the hospitalization was complicated by pseudomembranous enterocolitis. Tracheostomy insertion at six months of age enabled ongoing home ventilation.

**Figure 1:**
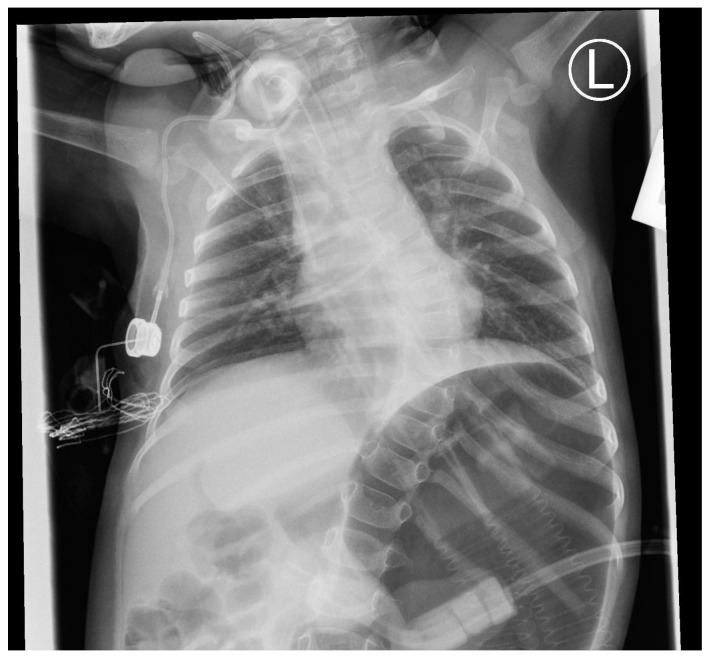
Features of gastroparesis and scoliosis in patient no. 1 at 13 months.

At 13 months, she required hospitalization to optimize nutritional management. Before admission, the girl was continuously fed via an intra-gastric tube and was experiencing increasing feeding difficulties, including frequent vomiting, regurgitation, and delayed gastric emptying (with 20–30 mL retention). Chronic constipation, intermittent tachycardia, and increased sweating were also reported. Upon physical examination, the girl was in a stable general condition, on ventilation via tracheostomy, and fed via a G-tube, with markedly reduced muscle tone, predominantly in the lower limbs. Moreover, the abnormal positioning of the right upper limb with internal rotation, varus foot positioning, left-sided scoliosis, and hands with visible atrophy of the small metacarpal muscles and accumulation of fatty tissue within the proximal phalanges (fatty pads) were noted. She was diagnosed with gastroparesis, which, together with existing feeding difficulties, led to the placement of a percutaneous endoscopic gastrostomy (PEG) (Figure 1). At the same time, subclinical hypothyroidism was diagnosed, and levothyroxine treatment was initiated.

**Figure 2:**
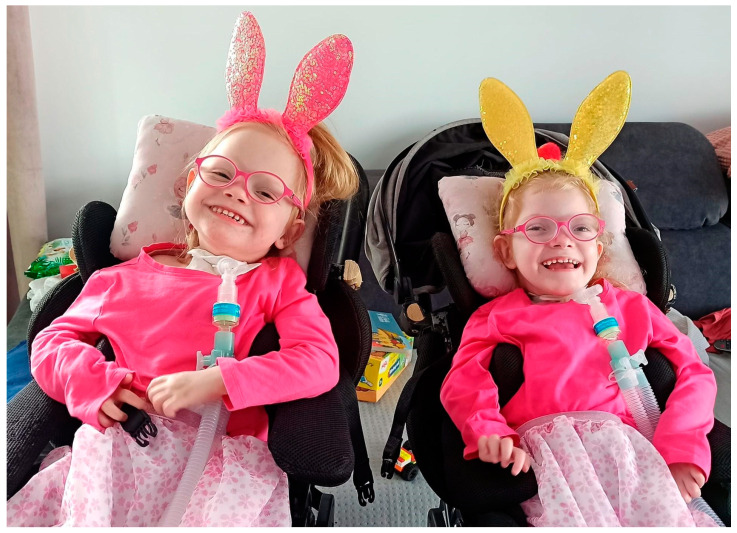
Patient no. 2 (**left**) and patient no. 1 (**right**) at the age of 4.5 years (courtesy of parents).

Currently, the girl is 4.5 years old and fed via a gastrojejunostomy tube inserted at the age of 3.5 years, requiring continuous respiratory support (Figure 2). At the age of 4 years, an ultrasound examination showed a completely paralyzed diaphragm. She has been receiving ongoing ophthalmic care since the age of four months due to astigmatism and requires ocular correction. The girl periodically presents neurovegetative symptoms in the form of episodes of gastroparesis (with abdominal pain, food retention, or persistent vomiting), constipation, a tendency toward tachycardia, sleep disorders, and neurogenic bladder. She is subjected to intensive physiotherapy. 

### 2.2. Patient No. 2. (Twin II)

The female infant was born prematurely at the 33rd week of gestation, as triplet III, via cesarean section. She was in fair general condition, with Apgar scores of 8/8/8, a birth weight of 880 g (−2.91 SD), a body length of 37 cm (−2.4 SD), and an OFC of 23 cm (−4.67 SD). She displayed features of morphological immaturity and FGR. After birth, the patient required respiratory support with infant flow and CPAP, surfactant administration, parenteral nutrition, and combined antibiotic therapy. Anemia was managed through multiple blood transfusions. Additionally, she was diagnosed with subclinical hypothyroidism and initiated on levothyroxine therapy at 2 weeks of age.

At 5 months of age, she was treated symptomatically for an RSV infection. By the age of 10 months, shortness of breath during sleep, deterioration in exercise tolerance, and episodes of desaturation during physiotherapy were observed. At 11 months, she required hospitalization in a PICU due to severe pneumonia and respiratory failure following yet another RSV infection. Head and chest computed tomography (CT) showed no significant abnormalities, including diaphragmatic paralysis. With rising difficulties in swallowing by 12 months of age, feeding via gastrointestinal tube was introduced. Episodes of tachycardia required the introduction of metoprolol therapy. During hospitalization, mechanical ventilation was initiated, ultimately leading to a tracheostomy at 13 months of age, followed by home ventilation.

Neurological, cardiological, and pulmonological work-ups were performed at 18 months of age. Despite ongoing care, she was solely fed with a GI tube and experienced restlessness, oxygen desaturation, tachycardia post-feeds, chronic constipation, and increased sweating. Globally decreased muscle tone, particularly in the head–torso axis, with an inability to sit independently and bilateral hand function by grasping and transferring objects were observed. Cranial nerve examination showed no abnormalities, although facial expressions were limited. Musculoskeletal evaluation indicated reduced muscle tone and strength, especially in the lower limbs, as well as scoliosis. Upper limb assessment revealed the atrophy of small metacarpal muscles and fatty pads. Talipes equinovarus and joint contractions were present. Respiratory assessment detected periodic wheezing. Neurological and orthopedic consultations recommended systematic physiotherapy and orthotic interventions to address musculoskeletal issues.

At the age of 21 months, she was hospitalized due to deepening difficulties with enteral feeding via nasogastric tube, resulting in a diagnosis of gastroparesis and subsequent placement of a PEG (Figure 2).

**Figure 3:**
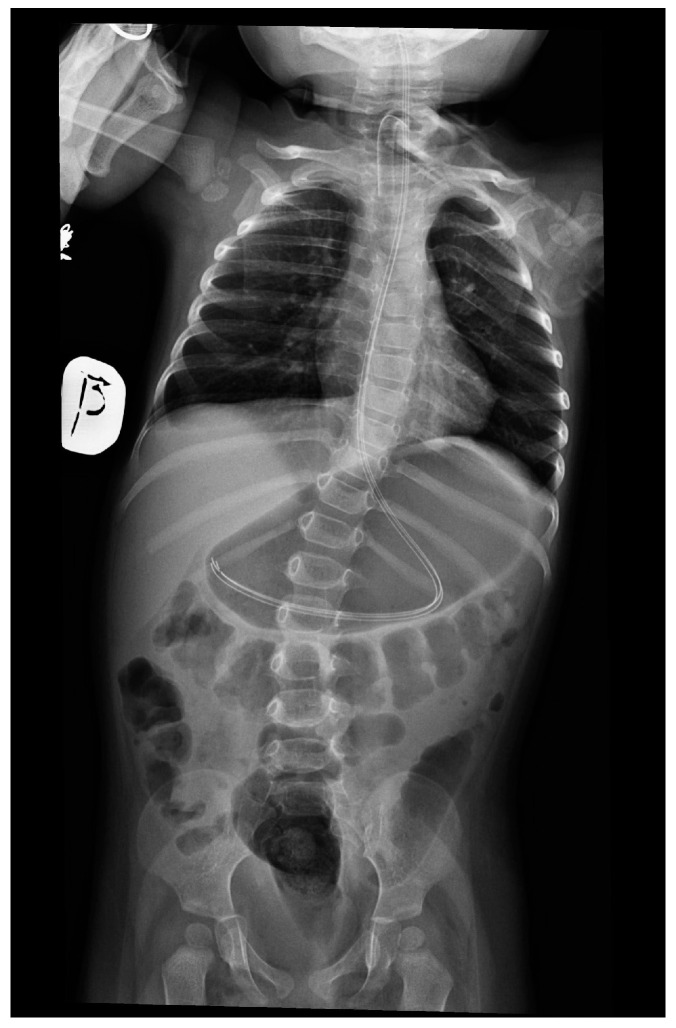
Features of gastroparesis and scoliosis in patient no. 2 at 18 months.

Currently, the girl is 4.5 years old, fed by a gastrostomy, and requires continuous respiratory support (Figure 3). At the age of 4 years, an ultrasound showed the paralysis of the right side of the diaphragm with a partially preserved function of the left side. Like her sister, she has been under ophthalmic care since the age of 4 months due to astigmatism and requires ocular correction. She also presents sleep disorders and symptoms of neurogenic bladder. The girl undergoes intensive physiotherapy.

The parents reported that since birth, both girls presented slower psychomotor development compared to their healthy brother.

#### Genetic Analysis

In both sisters (patient 1 and 2), during the hospitalization at the age of 14 months, a genetically determined neuromuscular disorder, primarily SMA, was taken into consideration (at this time, SMA was not included yet in the population newborn screening panel in Poland). A whole exome sequencing study (WES, Centogene^®^, Rostock, Germany) was performed on both girls, their brother, and their parents. The study identified in patients no. 1 and no. 2 two heterozygous likely pathogenic (LP) variants in the *IGHMBP2* gene: c.595G>C/p.Ala199Pro and c.1615_1623del/p.Ser539_Tyr541del. In their parents, carriership status was confirmed (mother: p.Ala199Pro variant; father: c.1615_1623del variant). Genetic diagnostics excluded a role for variants in activating cryptic splicing sites. One variant of *IGHMBP2* gene inherited from the mother was found in a healthy brother. No other suspected pathogenic or likely pathogenic variants were identified in the WES study. Based on the clinical evaluation and the results of molecular studies, the diagnosis of SMARD1 was confirmed in both affected children.

### 2.3. Patient No. 3

The female infant, the first child of unrelated healthy parents, was delivered at term via cesarean section with a birth weight of 2530 g (−1.68 SD), length of 50 cm (0.17 SD), and OFC of 31.5 cm (−2 SD). The Apgar’s assessment scored 10 points. Pregnancy was complicated by the lack of fetus weight gain since the 35th week of gestation. She was born with features of morphological immaturity and FGR. The neonatal period was uneventful, with proper body weight gain (750 g/month). However, starting from 2nd month of life, the girl exhibited a poor appetite and failure to thrive. She began to skip meals, displayed reluctance towards breastfeeding, and experienced a notable decline in weight gain to 300 g/month. Additionally, progressive muscle weakness, fatigue during meals, and abnormal foot positioning were observed.

At 3.5 months, she was admitted with gastroenteritis, receiving symptomatic treatment. At the end of hospitalization, she was transferred to the Department of Pediatrics and Neurology due to the worsening of the general condition, and grunting, significant respiratory effort, stridor, inspiratory chest positioning, diaphragmatic contraction, and hypotonia, especially in the head–torso axis, were observed. Additionally, her feeble crying raised concern. Neurological examination revealed globally reduced muscle tone with predominance in the lower limbs with the presence of symmetrical deep reflexes. A chest X-ray indicated an elevated position of the right diaphragm. A lung ultrasound examination identified slight paravertebral atelectasis, possibly attributable to shallow breathing.

At 4 months of age, respiratory arrest and bradycardia occurred during gastric tube insertion. Cardiopulmonary resuscitation (CPR) was successfully performed. Following a 10-day course of passive oxygen therapy, oxygen supplementation was no longer necessary, although transient drops in saturation to 88% were observed during crying spells.

**Figure 4:**
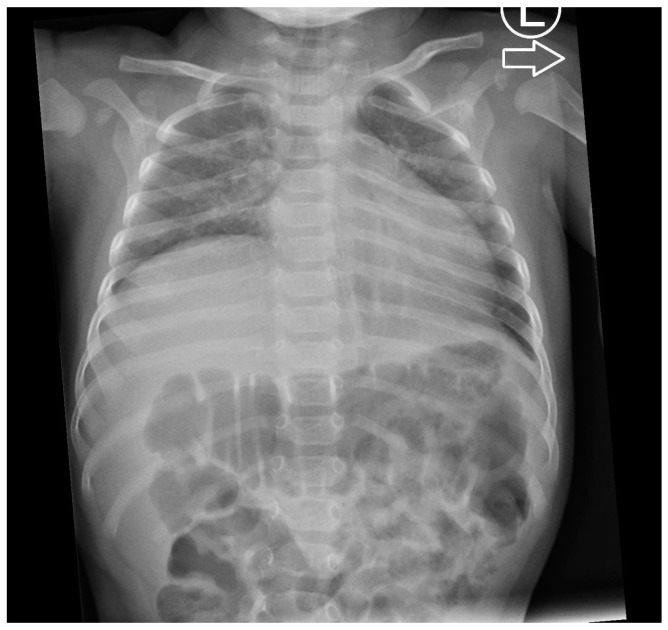
A chest X-ray of the patient no. 3 with right-sided eventration of the diaphragm at 4 months.

Upon admission to the pediatric department at the age of 4.5 months, the patient presented with a guarded general condition, with tachypnea (up to 60 breaths/minute) and tachycardia (ranging from 140 to 180 beats/minute). She exhibited considerable respiratory effort with a diaphragm contraction, adequate oxygenation, inspiratory chest positioning, grunting, and feeble crying. Parents pointed out their child’s decreased sensitivity to pain. Global hypotonia, particularly noticeable in the head–torso axis, and feet plantarflexion position were evident. A neurological examination revealed tongue fasciculations, a weak sucking reflex, decreased muscle tone in the upper and lower extremities with diminished deep tendon reflexes, and talipes equinovarus. In biochemical tests, no significant abnormalities were found. Echocardiography indicated the possibility of left ventricular hypertrophy. The brain and spinal canal MRI showed no significant abnormalities. No abnormalities in the larynx were detected. Throughout the hospitalization, the patient was fed orally and by gastric tube in a ratio of 30%:70%, achieving satisfactory weight gain (1000 g per month). Initially, due to the episodes of desaturation, passive oxygen therapy was introduced. A chest X-ray showed the eventration of the right hemidiaphragm (Figure 4). The symptoms of respiratory failure were continuously progressing, and at the age of 4.5 months, a tracheostomy was performed. This allowed for the patient to be discharged home for home ventilator therapy.

#### Genetic Analysis

The clinical evaluation of patient 3 was indicative of a genetically determined neuromuscular disorder, with a strong suspicion of SMARD1, which was finally confirmed by the WES examination.

WES Trio (Centogene^®^, Rostock, Germany) identified a heterozygous pathogenic variant c.439C>T/p.Arg147* and a heterozygous likely pathogenic variant c.1478C>T/p.Thr493Ile in the *IGHMBP2* gene, conclusively establishing the genetic diagnosis of SMARD1. In both families variants, compound heterozygous states were confirmed by the parental segregation. No other suspected disease-causing variants were identified in the WES study.

**Figure 5:**
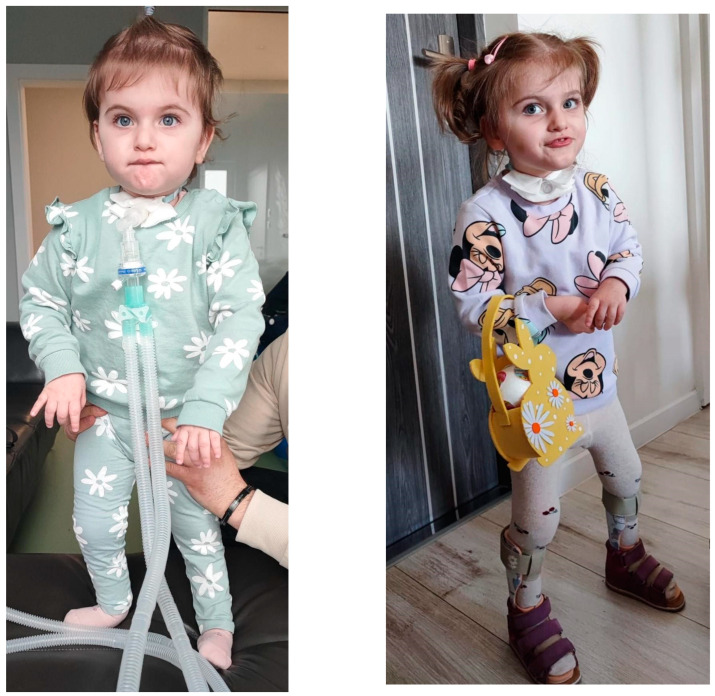
Patient no. 3 photographed at 1.5 years (**left**) and 2.5 years of age (**right**) (courtesy of parents).

The girl, who is currently 2.5 years old, is fed through a nutritional gastrostomy tube and needs respiratory support. She can go without ventilation while at rest for up to 2 h. Despite her disease, she is a very active child, undergoing intensive physiotherapy daily, and has been able to walk with a stabilization aid since the age of 1.5 years (Figure 5).

The selected characteristics of the patients are shown in Table 1.

## 3. Discussion

According to the available literature, in some patients with SMARD1, the onset of respiratory distress is triggered by a respiratory tract infection. Although both of the presented sisters had RSV infections at similar ages, only one of them (patient no. 1) developed complicated pneumonia with respiratory failure requiring mechanical ventilation at the PICU followed by constant home ventilation.

Moreover, the course of the disease in patient no. 1 seems to be more severe than in the two other children. In this patient, increased gastrointestinal symptoms (including gastroparesis) and difficulties in tolerating enteral feeding, which were not initially observed in the other two girls, are noteworthy. However, all patients presented symptoms of progressive muscle weakness, generalized hypotonia, fatty pads, limb contractures, progressive scoliosis, and thoracic deformity in the following months of life.

During the progression of SMARD1, the involvement of the sublingual nerve may occur, leading to tongue fasciculations. Grohmann et al. demonstrated these changes in around 30% of patients [7]. Tongue fasciculations were noted in patient no. 3 since the onset of symptoms. The dysregulation of the autonomic system is also a common feature of the disease. All children we reported had autonomic dysregulation features, which were more pronounced in the twins and included gastrointestinal symptoms and neurogenic bladder. Patient no. 3 demonstrated reduced pain perception. Other characteristic symptoms are joint contractures, primarily distal, which develop early and are likely secondary to muscle atrophy. Patients no. 1 and no. 2 also showed increased spinal deformities in the form of scoliosis, necessitating specialized corsets for stabilization and enhanced physiotherapeutic outcomes. Talipes equinovarus was present in all patients and worsened over time, requiring orthoses for management. Despite foot defects and progressive hypotonia, patient no. 3 is reportedly capable of assuming a standing position with orthotic support and assistance from others. Neither of the twins has achieved independent sitting or standing abilities.

In patients no. 1 and no. 2, the molecular analysis detected two variants in the *IGHMBP2* gene: c.595G>C/p.Ala199Pro and c.1615_1623del/p.Ser539_Tyr541del. The c.1615_1623del variant is an in-frame deletion of 9 bps in exon 11, which causes the loss of three residues. The c.595G>C variant causes an amino acid change from Ala to Pro at position 199. According to available molecular database information, these variants are classified as likely pathogenic. The variants have been described previously by Jędrzejowska et al., and both variants are considered likely pathogenic [21].

The p.Ala199Pro variant was identified in the mother and healthy brother (triplet I), while the c.1615_1623del variant was found in the father in a heterozygous state. Thus, it was confirmed that both parents carry variants in the recessive gene, indicating a compound heterozygous state in the patients.

It is noteworthy that initially, the described variants were classified as variants of unknown significance (VUS), but with the development of genetic studies and the expansion of genetic databases, the result was re-evaluated, changing the strength of the variant to likely pathogenic.

In patient no. 3, the WES analysis revealed two heterozygous variants in the *IGHMBP2* gene: the c.1478C>T/p.Thr493Ile variant and the c.439C>T/p.Arg147* variant. The c.1478C>T/p.Thr493Ile variant identified in the *IGHMBP2* gene is a missense variant, previously reported in the literature in association with SMARD1 [15,22]. A bioinformatics analysis suggests its potential pathogenicity. Additionally, the c.439C>T/p.Arg147* variant in the *IGHMBP2* gene, also documented in the literature in connection with SMARD1, is a pathogenic variant causing a premature STOP codon, thereby truncating the protein translation [21,23]. Confirmation of these mutations indicates the presence of a compound heterozygous state in the *IGHMBP2* gene, confirming the autosomal recessive mode of inheritance for the genetic alteration.

At the time of diagnosis, only patient no. 3 was found to have SMARD-specific diaphragmatic paralysis. In the 20 patients reported by Viguier et al. in 2019, 85% had diaphragmatic paralysis, in most cases right-sided [13]. In the majority of patients, diaphragmatic dysfunction is diagnosed simultaneously with the onset of the first symptoms of respiratory failure. However, cases of the late diagnosis of diaphragmatic palsy are described, as it was in the case of our twin sisters. Although hemidiaphragm eventration is a classic symptom of SMARD1, it may occur later in life, and therefore, its absence should not delay the inclusion of SMARD1 in the differential diagnosis in neonates and infants with respiratory failure [15]. In such cases, the urgent sequencing of the *IGHMBP2* gene is recommended. In the case of a normal *IGHMBP2* gene sequencing result, it is advisable to perform a test targeting intragenic and chromosomal rearrangements (e.g., Multiplex Ligation-dependent Probe Amplification test—MLPA—or array Comparative Genomic Hybridization—aCGH) to analyze gene deletion or duplication.

Performing genetic tests for SMARD1 also has limitations. According to the ClinVar database, due to the significant variability (polymorphism) of the *IGHMBP2* gene, the majority of described cases are classified as VUS, making it difficult to determine the pathogenicity of a particular variant [24]. Understanding the molecular and biochemical basis allows for attempts to create targeted therapies. Studies are underway to assess the clinical significance of described mutations as well as attempts to evaluate the functionality of the protein encoded by the *IGHMBP2* gene in vitro [11,12,20]. Currently, there is no causal treatment for SMARD1.

In the classic course of SMARD1, respiratory failure usually occurs between the 3rd and 12th month of age. In 2003, Pitt et al. proposed diagnostic criteria to help differentiate SMARD1 from other neurological diseases of early onset. These criteria include (1) low birth weight, the onset of the symptoms before 3 months of age, unilateral or bilateral diaphragmatic paralysis, the need for mechanical ventilation less than 4 weeks after the onset of sucking difficulties, and the exclusion of dysmorphia and other diseases; (2) characteristic changes found on histopathology of a peripheral nerve biopsy; and (3) evidence of acute denervation with the significant slowing of nerve conduction on electromyography (EMG) [25].

Our presented patients partly fulfilled the criteria outlined by Pitt et al. [25]; however, an invasive diagnosis (muscle biopsy, EMG) would have been necessary for a definitive diagnosis. With the increasing use of genetic tests, it seems reasonable to perform them at an early stage of diagnostics, as this minimizes the need for invasive procedures and significantly speeds up the establishment of the final diagnosis. In our patients, the diagnoses were based on clinical presentation and the results of the WES examination;, therefore the invasive neurophysiological tests were not justified.

Symptoms that may direct clinicians to the need for genetic testing for SMARD1 are the early diagnosis of respiratory failure of unknown etiology accompanied by muscle tone abnormalities. In recent years, an increasing number of diagnoses have been made based on prenatal molecular diagnosis, particularly in pregnancies complicated with FGR and reduced fetal movements. Investigations in this direction should be considered in families with cases of infant deaths in the course of respiratory failure of undetermined etiology or the course of sudden infant death syndrome (SIDS). In 2004, Chen et. al. presented a case of twins with SMARD 1, whose diagnosis was made prenatally due to a complicated family history [26].

The majority of children with SMARD1 are born prematurely and/or exhibit features of FGR. Both conditions carry potential short- and long-term complications [27]. Prematurely born children often display symptoms of respiratory distress syndrome (RDS), which could impact their pulmonary function in the future. However, there is insufficient data to determine whether RDS or other pulmonary diseases are risk factors for the earlier onset of SMARD1 symptoms. Two of our patients (no. 1 and no. 2) experienced multiple complications of prematurity, including respiratory distress, anemia, and infection. These factors alone could affect their development independent of the underlying neurological disorder. While some long-term complications of FGR may not directly apply to SMARD1 patients (e.g., short stature), as lifespan increases, consideration must be given to other comorbidities unrelated to the primary disorder (such as hypothyroidism or adrenarche praecox) [28,29].

Numerous authors emphasize the diverse symptomatology observed in patients with variants in the *IGHMBP2* gene. Symptoms range from a classical course of SMARD1 to those presenting with late-onset or ones resembling CMT2S. CMT2S typically presents as an autosomal recessive axonal neuropathy, usually beginning in the first decade of life with a gradual onset of muscle weakness and wasting in the distal regions of the upper and lower limbs. Patients often display diminished reflexes and varying degrees of sensory loss in the distal areas. While this condition should be considered in patients exhibiting progressive muscle weakness, it is noteworthy that diaphragmatic paralysis and secondary respiratory failure are not features of CMT2S.

One of the diseases that should be differentiated from SMARD1 is SMA. Nowadays, newborn screening for SMA is conducted in numerous countries worldwide, including Poland. This aids in streamlining the diagnostic process, particularly given the similar clinical presentation of SMA and SMARD1 stemming from damage to alpha-motoneurons in the spinal cord anterior horns [18]. A comparative description of the conditions has been presented in Table 2. Despite their similarities, these conditions arise from variants in different genes. The most common mutation responsible for SMA is a homozygous deletion of *SMN1* exon 7, easily identifiable and utilized as a sensitive diagnostic marker [30]. Up to now, three therapeutical options for SMA patients have received approval [30]. Due to its rarity, the polymorphism of the *IGHMBP2* gene, and the absence of specific treatment, SMARD1 is not included in newborn screening programs.

In the cases published in the literature, each patient exhibits a slightly varied disease progression. The consistent feature is respiratory failure necessitating mechanical ventilation alongside progressive muscle weakness, primarily affecting distal regions. Efforts have been made to establish correlations between phenotype and genotype [13]. It has been suggested that the disease may manifest more severely in homozygotes than compound heterozygotes, with respiratory failure occurring earlier in these cases, although conclusive evidence is lacking. SMARD1 remains a heterogeneous condition.

## 4. Conclusions

Our work emphasizes the heterogeneity of clinical symptoms in SMARD1 individuals, even among patients with the same genotype. Moreover, we highlight the necessity of early genetic diagnostics in patients with respiratory failure with or without diaphragmatic involvement to differentiate muscle atrophy conditions. Our example of the clinical presentation of genetic variants previously considered as VUS emphasizes the value of collecting clinical data together with genetic findings to expand our understanding of the genetic disorders.

## Figures and Tables

**Table 1:** Selected characteristics of presented patients. FGR—fetal growth restriction. (“+”—present, “-“—not present).

	Patient No. 1 (Twin I)	Patient No. 2 (Twin II)	Patient No. 3
Sex	F	F	F
Age of onset of first symptoms [months]	4	11	2
Variant coordinates in *IGHMBP2* gene	c.595G>C/p.Ala199Pro; c.1615_1623del/p.Ser539_Tyr541del		c.439C>T/p.Arg147*; c.1478C>T/p.Thr493Ile
Symptoms			
FGR	+	+	+
Premature birth (gestational age [weeks])	+ (33)	+ (33)	- (39)
Feeding difficulties/dysphagia	+	+	+
Age of gastrostomy insertion [months]	13	21	4
Feeble crying	+	+	+
Site of the eventration of the diaphragm (age of diagnosis)	bilateral (4 years)	right (4 years)	right (3 months)
Onset of respiratory failure [months]	4	11	3
Age of tracheostomy insertion [months]	6	13	4
Ability to sit or walk assisted	-	-	+
Neurological evaluation	Globally reduced muscle tone predominantly in the lower limbs, talipes equinovarus, contractures of the distal joints, progressive muscle weakness		Globally reduced muscle tone predominantly in the central axis, contractures of the distal joints, talipes equinovarus, lack of deep tendon reflexes in the lower limbs, progressive muscle weakness, tongue fasciculations
Fatty pads	+	+	+
Scoliosis	+	+	-
Autonomic dysregulation	Gastroparesis, constipation, transient tachycardia, excessive sweating, neurogenic bladder	Transient gastroparesis, constipation, tachycardia, excessive sweating, neurogenic bladder	Decreased pain sensitivity
Comorbidities	Hypothyroidism	Hypothyroidism	-

**Table 2:** A comparison of the main features of spinal muscular atrophy with respiratory distress type 1 (SMARD1) and spinal muscular atrophy (SMA5q with subtypes) (“+”—present, “-“—not present) [30,31].

Feature	SMARD1	SMA5q
Gene	*IGHMBP2* (11q13)	*SMN1* (5q13.2)
Protein	IGHMBP2	SMN
Inheritance	Autosomal recessive	Autosomal recessive
Onset of symptoms (type of the disease) [months]	<3 (early onset)	Birth–6 (SMA1)
	3–12 (classical)	6–18 (SMA2)
	>12 (late onset)	>18 (SMA3)
		20–40 years (SMA4)
Pathophysiology	Motor neuron degeneration due to IGHMBP2 deficiency	Motor neuron degeneration due to SMN deficiency
Muscle weakness distribution	Distal	Proximal(lower limbs > upper limbs)
Joint contractures	+ (primarily distal)	+ in SMA1, SMA2, SMA3
Scoliosis	−	+ in SMA1, SMA2, SMA3
Deep tendon reflexes	−	−
Tremor	Tongue fasciculations	Tongue fasciculations; hand tremor in SMA3 and SMA4
Ventilatory impairment	+	+ in SMA1 and SMA2, rare in SMA3
Autonomic dysfunction	+	+
Modifier Gene	None	SMN2 copy number

## Data Availability

The original contributions presented in the study are included in the article, further inquiries can be directed to the corresponding author.
